# Regenerative role of mast cells and mesenchymal stem cells in histopathology of the sciatic nerve and tibialis cranialis muscle, following denervation in rats

**DOI:** 10.22038/ijbms.2024.78732.17030

**Published:** 2024

**Authors:** Zahra Bakhtiary, Rasoul Shahrooz, Rahim Hobbenaghi, Saeed Azizi, Farhad Soltanalinejad, Ali Baradar Khoshfetrat

**Affiliations:** 1 Department of Basic Sciences, Faculty of Veterinary Medicine, Urmia University, Urmia, Iran; 2 Department of Histology, Faculty of Veterinary Sciences, Ilam University, Ilam, Iran; 3 Department of Pathobiology, Faculty of Veterinary Medicine, Urmia University, Urmia, Iran; 4 Department of Surgery and Diagnostic Imaging, Faculty of Veterinary Medicine, Urmia University, Urmia, Iran; 5 Department of Chemical Engineering, Sahand University of Technology, Tabriz, Iran

**Keywords:** Denervation, Histology, Mast cells, Mesenchymal stem cells, Muscle, Rat, Regeneration

## Abstract

**Objective(s):**

Atrophy of the muscles following denervation can lead to the death of myofibers. This study evaluated the sciatic nerve and tibialis cranialis muscle (TCM) regeneration using scaffold and cells.

**Materials and Methods:**

Ninety adult male Wistar rats were divided into six main groups (n=15) and three subgroups (2, 4, and 8 weeks). Groups: control; without surgery, Tr; sciatic nerve transected in silicone tube, S; collagen gel put inside the silicone tube, MC; placed 3×10^4^ mast cells mixed with scaffold, MSC; placed 3×10^4^ mesenchymal stem cells mixed with scaffold, and MC+MSC; 3×10^4^ of each of the mast cell and mesenchymal stem cells along with scaffold. Animals were euthanized and sampled for muscle and nerve histological and nerve immunohistochemical evaluations.

**Results:**

Diameter of muscle fibers, ratio of the muscle fiber’s nuclei to the fibrocyte nuclei (mn/fn), ratio of the muscle fibers nuclei number to the muscle fiber’s number (mn/mf), and ratio of the blood vessels number to the number of muscle fibers (v/mf) in all treatment groups, especially the MC + MSC group, increased compared to the Tr group but the number of mast cells, the percentage of sarcoplasmolysis, and necrosis fibers decreased. Histomorphometric results of the nerve in its various parts and immunohistochemistry results also showed improved nerve conduction, especially in the MC + MSC group.

**Conclusion:**

In this study, nerve improvement happened mainly for two reasons: cells and time. So, the most obvious improvement occurred in the group with mast and mesenchymal cells in the 8^th^ week.

## Introduction

Every year, huge expenses are spent for the treatment of patients with peripheral nerve injuries, which in many cases lead to paralysis due to the extent of the injuries or the lack of appropriate treatment methods. Sciatic nerve transection is one of the most common models used to examine nerve and especially muscle lesions in rats (1).

Impairment of sensory and motor functions as well as muscle atrophy occurs immediately after denervation (2) and if not repaired leads to loss of muscle fibers and irreversible damage to the muscles under denervation (3). The onset of denervated muscle injury is associated with rapid loss of function, weight loss, and atrophy of muscle fibers (lasts 2 months). From 2 to 7 months it is characterized by severe atrophy and loss of sarcomere structures and in the last stage (from 7 months onwards), fibrosis of interstitial tissue and structures of adipose tissue with a sharp decrease in the number of muscle fibers is visible (4). In rodent models, muscle atrophy occurs within 1 to 2 weeks after denervation, and in a study performed on denervated posterior limbs of female rats, atrophy occurred within 14 days (5). The tibialis cranialis muscle (TCM) is one of the most widely used anterior-lateral muscles due to its availability and direct innervation by the sciatic nerve, which has always been considered by many researchers. One of the newest and most efficient methods of sciatic nerve regeneration is the use of natural or synthetic scaffolds along with cells capable of secreting nerve growth factors at the site of nerve transection by tissue engineering (6). Mast cells are cells that are scattered throughout all tissues. In addition to their essential role in the immune system against pathogens and allergens (7), these cells have a two-way relationship with the nerves and stimulate nerve growth with their secretions (8). This interaction between mast cells and nerves and the secretion of neuropeptides by mast cells (9) on the other hand triggers the mechanisms of nerve tissue regeneration following injury (10). Recent studies also have shown the beneficial effects of mesenchymal cells on nerve regeneration (11). In addition, they can differentiate into neuron-like Schwann cell types *in vivo *and* in vitro* (12). Mast cells also increase the proliferation and transport of mesenchymal stem cells (13). In order to perform their neurogenic activities, cells are placed in an environment with physiological, biochemical, and biophysical conditions similar to an extracellular matrix called a scaffold (14). More recently, collagen microfibers (20 to 30 μm thick) have been used for relative performance recovery in experimental models of CNS and PNS injuries (15), and healing of neurological injuries has been proven by collagen gel (16). For example, a study of damaged nerve tissue found that neuronal regeneration performed well when tubular conduits were filled with materials such as collagen fibers and collagen gels (17).

The present study aimed to evaluate the improvement of the sciatic nerve and TCM following denervation using mast cells and mesenchymal cells by tissue engineering.

## Materials and Methods

***Experimental design and animals***

Ninety adult male Wistar rats weighing 150–200 g were randomly divided into six main groups (n=15) and each into three subgroups (n=5) in three time periods (2, 4, and 8 weeks). Animals were housed at 23 ± 3 °C, 12-hour light/dark cycle, with free access to standard rodent laboratory food and water, and were kept for 2 weeks before surgery. The groups comprised control, transection (Tr), scaffold (S), mast cell (MC), mesenchyme (MSC), and mast cell-mesenchyme (MC+MSC). The surgeries were performed under anesthesia and at least injury to the animals.

***Surgical procedure***

All procedures were carried out according to the guidelines of the Veterinary Ethics Commission of the Veterinary Faculty of Urmia University, Urmia, Iran (Reference No.: IR-UU-AEC-3/1656/AD). Animals were anesthetized by intraperitoneal administration of ketamine hydrochloride 5%, 90 mg kg^-1^ (Ketaset 5%; Alfasan, Woerden, The Netherlands) and xylazine hydrochloride 2%, 5 mg kg^-1^ (Rompun 2%, Bayer, Leverkusen, Germany) in sterile conditions (18). Surgery was performed in the control group without nerve transection, but in the other groups, a complete left sciatic nerve transection was created. For nerve transection, at first, the left sciatic nerve was exposed and fixed by silk ligature on epineurium to silicone conduit. Then transected and the free (distal) end was ligated on the other side of the conduit with an 8 mm distance between the two ends. In the Tr group, only the nerve was transected but in the S group, collagen-gel scaffold (10 μl), in the MC group, 3 × 10^4^ mast cells along with the scaffold, in the MSC group 3×10^4^ mesenchymal cells along with scaffold, and MC+MSC group 3×10^4 ^mast cells along with mesenchymal cells for each in scaffold were added to silicone tube. After sciatic nerve surgery, the muscle was sutured with 4/0 Vicryl (Ethicon, Norderstedt) and the skin with 3/0 nylon (Dafilon, B/Braun, Germany). 

***Histological analysis***

**Figure 1:**
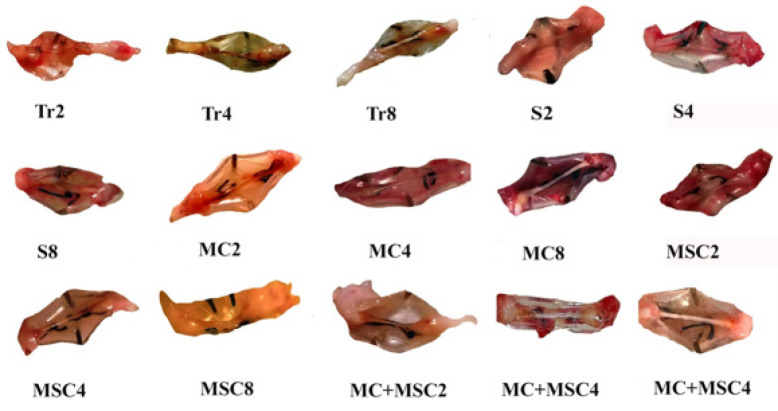
Macroscopic images of sciatic nerve repair inside silicone tubes in different groups of rats

Animals were euthanized at 2, 4, and 8 weeks after surgery using an overdose of ketamine-xylazine (90 mg/kg of 5% ketamine and 5 mg/kg of 2% xylazine) three times the anesthetic dose (IP). The sciatic nerve has been sampled from proximal, location, and distal parts of the transaction region (Figure 1), and a sample of tibialis cranialis muscles was prepared and fixed in the 10% formal saline solution and paraffin sections (5–7 μm) prepared by using a rotary microtome (Microm, GmbH, Germany). Then they were stained with hematoxylin-eosin (H&E) for histomorphometric studies. 

***Isolation and culture of mice mast cells***

According to a method previously described (19), Mast cells were derived from the bone marrow of male mice. The purity percentage of obtained mast cells was >90% by flow cytometry (20). 

*Isolation of mesenchymal stem cells from rats*

MSC cells were obtained from the rat’s femur and tibiae bone marrow based on a proven method (21). Mesenchymal stem cell purity >90% was demonstrated by flow cytometry (20).

***Histomorphometric studies of tibialis cranialis muscle***

1) Diameter of muscle fibers measured randomly in 20 fibers from each tissue sample using a calibrated graded objective lens. 

2) Average number of the muscle fiber’s nuclei ratio to the fibrocystic nuclei (mn/fn): counted in a fixed microscopic field (0.0625 mm^2^).

3) Average number of the muscle fibers nuclei ratio to the muscle fiber’s number (mn/mf): in a fixed number of muscle fibers cross sections (10 pcs) in each tissue sample.

4) Average number of blood vessels ratio to muscle fibers number (v/mf): in a fixed number of muscle fibers cross sections (10 pcs) in each tissue sample stained with the Masson Trichrome method. 

5) Mean number of mast cells: counting in a fixed microscopic field (0.0625 mm^2^) stained with the toluidine blue method.

6) Mean percentage of sarcoplasmolysis muscle fibers: counted in a fixed microscopic field (0.0625 mm^2^).

7) Mean percentage of necrotic muscle fibers: counted in a fixed microscopic field (0.0625 mm^2^).

***Histomorphometric studies of sciatic nerve***

**1) **Proximal sections;

The mean ratio of the Schwann cells nuclei number to the fibrocytic nuclei (scn/fn): counting in a fixed microscopic field (0.0625 mm^2^).

2) Site of nerve transection sections;

a) Mean ratio of the Schwann cells nuclei number to the fibrocytic nuclei (scn/fn): counting in a fixed microscopic field (0.0625 mm^2^).

b) Mean percentage of myelin fibers: calculated in a fixed microscopic field (0.0625 mm^2^) stained with the Luxol fast blue-crystal violet method.

*Nerve fascicle formation was also examined at the site of nerve transection.

3) Distal sections;

a) Mean ratio of the Schwann cell nuclei number to the fibrocytic nuclei (scn/fn): counting in a fixed microscopic field (0.0625 mm^2^).

b) Average of spheroid fibers number: counting in a fixed microscopic field (0.0625 mm^2^).

c) Average number of mast cells: counting in a fixed microscopic field (0.0625 mm^2^) that stained with the Toluidine blue method.

d) Mean percentage of myelin fibers: calculated in a fixed microscopic field (0.0625 mm^2^) stained with the Luxol fast blue-crystal violet method. 

e) Average number of myelin fibers: counting in a fixed microscopic field (0.0625 mm^2^) stained with the Luxol fast blue-crystal violet method.

f) Average diameter of perineurium thickness to the nerve fascicle thickness ratio (peri/fas): It was measured by using a calibrated graded objective lens in a fixed microscopic field (1 mm^2^). 

*Furthermore, the presentation of S-100 protein in distal nerve sections by immunohistochemistry (IHC) was measured. 

***Collagen gel scaffold***

Collagen gel was obtained from SBPE Company (Tabriz, Iran).

***Statistical analysis***

Data were analyzed by SPSS software (version 20, SPSS Inc., Chicago, IL, USA), one-way ANOVA, and Tukey *post hoc* test. Results were reported as mean ± SE and significant differences between experimental groups were set at *P*<0.05. 

## Results

***Morphometrical results of tibialis cranialis muscles in different groups and time periods***

*Diameter of muscle fibers*

The mean diameter of muscle fibers in the second week showed a significant decrease in the Tr group compared to the other treatment groups (*P*<0.05). In the fourth week, the Tr group was significantly different from the control and S groups (*P*<0.05). Also, in the 8th week, all treatment groups had the highest diameter and only the MC + MSC group showed a significant increase compared to the control and Tr groups (*P*<0.05). (Table 1).

*Ratio of the number of muscle fiber nuclei to the number of fibrocyte nuclei (mn/fn)*

The average of this ratio in the second week showed a significant decrease in the Tr group compared to the control group (*P*<0.05), but in the S and cell receiving groups, although there was an increase in the ratio, there was no significant difference with the Tr group. In the 4^th^ week, the lowest ratio was observed in Tr groups which was significantly different from other groups (*P*<0.05). Also, the highest mean ratio was observed in the control and MC + MSC groups. In the 8th week, there was a significant decrease in (mn/fn) in the Tr group compared to all groups (*P*<0.05) (Table 1).

**Table 1:** Comparison of histomorphometric parameters of tibialis cranialis muscle (TCM) between the tested groups and three different times for each grou of rats (Mean ± Se)

Groups		Control				Tr				S				MC				MSc			MC+MSc				
Weeks	2		4	8	2		4	8	2		4	8	2		4	8	2		4	8		2	4	8
Number of muscle mast cells	1.10 ± 0.19^a^		1.15 ± 0.20^a^	1.05 ± 0.19^a^	15.50 ± 0.81^e^ ×		15.25 ± 0.64^d^ ×	10.8 ± 1.04^c^ ≠	13.05 ± 1.02^de^ *		12.35 ± 1.04^c^ *	7.35 ± 1.16^b^ ≠	10.25 ± 0.99^bc^ ×		10.00 ± 0.98^bc^ ×	6.60 ± 1.10^b^ ≠	11.15 ± 1.11^cd^ *		12.10 ± 0.98^c^ *	7.05 ± 1.08^b^ ≠		8.25 ± 1.02^b^ ×*	9.20 ± 0.94^b^ *	5.55 ± 1.15^b^ ×
Sarcoplasmolysed muscle fibers %	-		-	-	18.44 ± 0.97^b^ ×		24.33 ± 1.30^c^ *	13.22 ± 0.70^d^ ≠	16.33 ± 0.81^ab^ ×		17.44 ± 0.81^ab^ ×	10.44 ± 0.50^c^ ≠	14.22 ± 0.86^a^ *		16.33 ± 1.04^ab^ *	7.88 ± 0.78^b^ ≠	17.33 ± 1.19^b^ ×		18.33 ± 0.78^b^ ×	8.88 ± 0.58^bc^ ≠		13.77 ± 0.92^a^ *	15.33 ± 0.62^a^ ×	3.88 ± 0.73^a^ ≠
necrotic muscle fibers %	-		-	-	24.11 ± 1.33^c^ *		24.66 ± 1.59^c^ *	15.88 ± 0.67^d^ ≠	20.11 ± 1.27^b^ ×		18.88 ± 1.38^b^ ×	12.77 ± 0.84^c^ ≠	19.55 ± 0.86^b^ *		19.33 ± 0.92^b^ *	8.11 ± 0.69^ab^ ≠	18.33 ± 0.88^b^ ×		18.44 ± 0.92^b^ ×	9.88 ± 0.61^b^ ≠		12.88 ± 0.78^a^ *	12.22 ± 0.86^a^ *	6.66 ± 0.62^a^ ≠

The letters are for comparison between groups at any time (week 2 green, week 4 red, and week 8 black), and the symbols (×, *, ≠) are for comparison between three different times in each group. The presence of non-similar letters or symbols indicates a significant difference (*P*<0.05)

**Table 2:** Comparison of histopathologic parameters of TCM between the tested groups and three different times for each group of rats (Mean ± Se)

Groups		Control				Tr			S			MC			MSc			MC+MSc		
Weeks	2		4	8	2		4	8	2	4	8	2	4	8	2	4	8	2	4	8
Diameter of muscle fibers (µm)	37.9 ± 0.16^c^ ×		37.5 ± 0.17^c^ ×	36.6 ± 0.20^c^ ×	14. 7 ± 0.08^a^ *		15 .0 ± 0.13^a^ *	22.6 ± 0.12^a^ ≠	18.8 ± 0.08^b^ ×	19.0 ± 0.10^b^ ×	22.9 ± 0.08^a^ ≠	21.6 ± 0.09^b^ ×	15.5 ± 0.07^ab^ ≠	25.9 ± 0.14^ab^ *	19.6 ± 0.11^b^ ≠	15.2 ± 0.12^ab^ *	22.4 ± 0.12^a^ ≠	21.9 ± 0.12^b^ *	18.5 ± 0.13^ab^ *	28.5 ± 0.14^b^ ×
mn/mf	1.98 ± 0.15^b^		1.71 ± 0.09^d^	1.94 ± 0.08^c^	1.04 ± 0.02^a^ *		0.95 ± 0.04^a^ *	1.16 ± 0.01^a^ ≠	1.23 ± 0.05^a^ ×	1.22 ± 0.05^bc^ ×	1.42 ± 0.05^b^ ≠	1.28 ± 0.06^a^ *	1.13 ± 0.02^b^ ≠	1. 34 ± 0.04^b^ *	1.23 ± 0.05^a^ ×	1.18 ± 0.03^b^ ×	1. 37 ± 0.03^b^ ≠	1.23 ± 0.05^a^ *	1.36 ± 0.04^c^ ≠	1.48 ± 0.03^b^ ≠
mn/fn	1.56 ± 0.05^c^		1.43 ± 0.07^d^	1.52 ± 0.05^c^	1.03 ± 0.01^a^ *		1.06 ± 0.02^a^ *	1.25 ± 0.03^a^ ≠	1.26 ± 0.01^b^ ×	1.27 ± 0.03^ab^ ×	1.35 ± 0.02^ab^ ≠	1.21 ± 0.03^b^ *	1.21 ± 0.01^bc^ *	1.37 ± 0.03^ab^ ≠	1.22 ± 0.03^b^ ×	1.16 ± 0.01^bc^ ×	1.39 ± 0.02^a^ ≠	1.31 ± 0.03^b^ *	1.28 ± 0.02^c^ *	1.45 ± 0.03^a^ ≠
V/mf	1.40 ± 0.03^d^		1.45 ± 0.03^d^	1.43 ± 0.04^e^	0.63 ± 0.01^a^ ×		0.73 ± 0.01^a^ *	0.93 ± 0.01^a^ ≠	0.92 ± 0.01^b^ *	0.95 ± 0.02^bc^ *	1.09 ± 0.02^bc^ ≠	0.99 ± 0.01^c^ ×	0.92 ± 0.01^b^ *	1.15 ± 0.01^cd^ ≠	0.95 ± 0.01^bc^ *	0.93 ± 0.01^b^ *	1.05 ± 0.03^b^ ≠	1.00 ± 0.02^c^ ×	1.01 ± 0.03^c^ ×	1.23 ± 0.03^d^ ≠

The letters are for comparison between groups at any time (week 2 green, week 4 red, and week 8 black), and the symbols (×, *, ≠) are for comparison between three different times in each group. The presence of Nonnon-similar letters or symbols indicates a significant difference (*P*<0.05)

**Figure 2:**
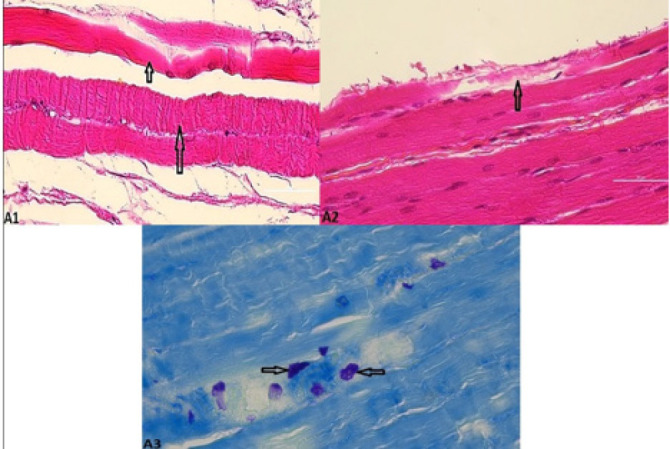
A1) Microscopic images of eosinophilic, curly (large arrows), and necrosis muscle fibers (small arrows), H&E staining, 400× A2) Sarcoplasmolyzed muscle fibers (arrows). H&E staining, 400× A3) Mast cells containing metachromatic granules between muscle fibers (arrows), toluidine blue staining 400×

**Table 3:** Comparison of histomorphometric parameters of the sciatic nerve (proximal sections, transection site) between the tested groups and three different times for each group of rats (Mean ± Se)

Groups		Control					Tr			S				MC				MSc			MC+MSc			
Weeks	2		4	8	2	4		8	2		4	8	2		4	8	2		4	8		2	4	8	
scn/fn in the proximal nerve	2.04 ± 0.08^b^		1.98 ± 0.04^b^	2.03 ± 0.03^b^	1. 59 ± 0.03^a^ *	1. 59 ± 0.02^a^ *		1. 64 ± 0.02^a^ *	1. 62 ± 0.02^a^ ×		1. 62 ± 0.01^a^ ×	1. 66 ± 0.01^a^ ×	1. 63 ± 0.02^a^ *		1. 63 ± 0.02^a^ *	1. 65 ± 0.01^a^ *	1. 62 ± 0.01^a^ ×		1. 61 ± 0.02^a^ ×	1. 67 ± 0.01^a^ ≠		1. 61 ± 0.02^a^ *	1. 66 ± 0.01^a^ *×	1. 69 ± 0.01^a^ ×	
scn/fn at the site of nerve transection	-		-	-	1.18 ± 0.04^a^ *	1.29 ± 0.03^a^ *		1.47 ± 0.06^a^ ≠	1.39 ± 0.02^b^ ×		1.41 ± 0.02^b^ ×	1.62 ± 0.04^b^ ≠	1.39 ± 0.06^b^ *		1.39 ± 0.02^b^ *	1.75 ± 0.02^bc^ ≠	1.40 ± 0.01^b^ ×		1.44 ± 0.03^b^ ×	1. 68 ± 0.04^b^ ≠		1.50 ± 0.02^b^ *	1.56 ± 0.01^c^ *	1. 89 ± 0.06^c^ ≠	
% myelinated fibers at the site of nerve transection	-		-	-	37.00 ± 1.26^a^ *	36.00 ± 2.28^a^ *		45.60 ± 0.92^a^ ≠	39.20 ± 2.22^a^ ×		39.60 ± 2.44^a^ ×	50.20 ± 1.24^b^ ≠	39.40 ± 1.91^a^ *		39.60 ± 1.07^a^ *	50.20 ± 1.15^b^ ≠	38.60 ± 1.77^a^ ×		40.20 ± 0.96^a^ ×	50.00 ± 0.89^b^ ≠		41.60 ± 1.50^a^ *	41.60 ± 1.46^a^ *	52.00 ± 1.00^b^ ≠	

The letters are for comparison between groups at any time (week 2 green, week 4 red, and week 8 black), and the symbols (×, *, ≠) are for comparison between three different times in each group. The presence of non-similar letters or symbols indicates a significant difference (*P*<0.05)

**Figure 3:**
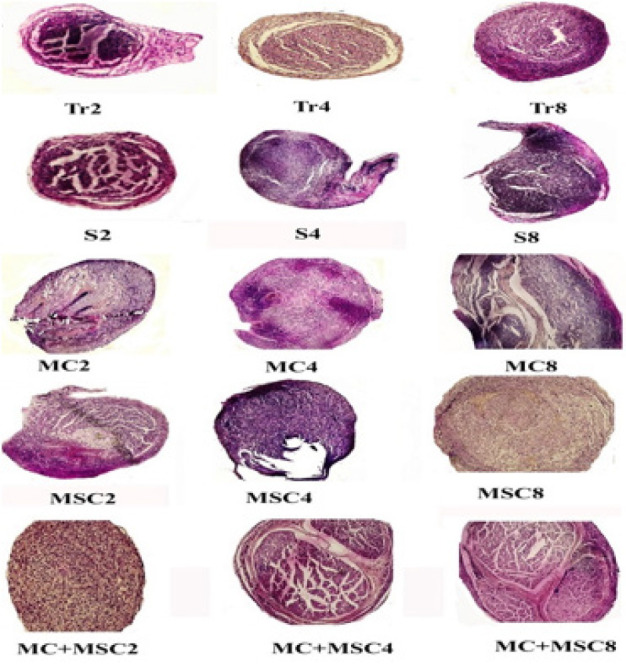
Nerve trunk formed at the site of sciatic nerve transection in different groups of rats H&E staining 100×

**Table 4:** Comparison of histomorphometric parameters of the sciatic nerve in the distal part of the transection site between the groups and three different times for each group of rats (Mean ± Se)

Groups		Control				Tr				S			MC			MSc			MC+MSc		
Weeks	2		4	8	2		4	8	2		4	8	2	4	8	2	4	8	2	4	8	
scn/fn	2.04 ± 0.08^d^		1.98 ± 0.04^d^	2.03 ± 0.03^e^	1.45 ± 0.04^a^ *		1.39 ± 0.04^a^ *	1.60 ± 0.02^a^ ≠	1.51 ± 0.02^ab^ ×		1.56± 0.03^b^ ×	1.70 ± 0.01^b^ ≠	1.62 ± 0.03^bc^ *	1.61± 0.02^bc^ *	1.77 ± 0.01^cd^ ≠	1.62 ± 0.03^bc^ ×	1.61 ± 0.01^bc^ ×	1.73 ± 0.01^bc^ ≠	1.66 ± 0.01^c^ *	1.69± 0.02^c^ *	1.80± 0.01^d^ ≠	
spheroid nerve fibers	-		-	-	53.97± 2.68^c^ *		57.71± 3.41^c^ *	41.88± 1.59^d^ ≠	46.96 ± 2.72^b^ ×		43.34± 2.07^b^ ×	36.97± 0.96^c^ ≠	40.32 ± 1.93^a^ *	37.91± 1.41^ab^ *	32.36± 0.69^b^ ≠	41.25 1.86^ab^ ×	39.86± 1.67^ab^ ×	33.81 1.03^bc^ ≠	36.37 ± 0.61^a^ *	35.93± 1.05^a^ *	29.03± 1.09^a^ ≠	
Number of mast cells	1.30 ± 0.33^a^		1.20 ± 0.29^a^	1.30 ± 0.30^a^	10.70± 0.73^d^ *		9.80 ± 0.62^c^ *	6.70 ± 0.36^d^ ≠	8.00 ± 0.36^bc^ ×		7.90± 0.50^b^ ×	5.00 ± 0.47^c^ ≠	7.40 ± 0.54^bc^ *	6.80 ± 0.59^b^ *	4.80 ± 0.41^c^ ≠	8.50 ± 0.50^c^ ×	7.80 ± 0.38^b^ ×	6.00 ± 0.59^cd^ ≠	6.77 ± 0.47^b^ *	6.70± 0.36^b^ *	2.90± 0.23^b^ ≠	
Myelinated fibers %	90.8 ± 2.15^b^		90.8 ± 2.15^b^	90.0 ± 1.41^b^	66.4 ± 2.71^a^ *		66.4 ± 1.93^a^ *	76.0 ± 2.00^a^ ≠	68.4 ± 2.13^a^ *×		67.2± 1.85^a^ *	74.0 ± 1.41^a^ ×	68.0 ± 1.41^a^ *	68.8 ± 2.41^a^ *	77.0 ± 1.32^a^ ≠	66.8 ± 1.85^a^ ×	67. 2 ± 1.62^a^ ×	75.0 ± 2.03^a^ ≠	68.4 ± 2.13^a^ *	68.4± 2.03^a^ *	78.0± 1.01^a^ ≠	
Myelinated fibers per microscopic surface unit	335.1 ± 6.85^a^		335.6 ± 7.55^a^	335.5 ± 7.38^a^	587.7± 35.3^b^ *		531.1± 45.7^b^ ×*	477.2± 20.6^c^ ×	632.8 ± 26.0^b^ *		641.3± 20.0^c^ *	410.5± 11.6^b^ ≠	618.3 ± 35.1^b^ ×	667.2± 28.3^c^ ×	356.7± 14.9^a^ ≠	612.7± 21.0^b^ *	634.5± 23.1^c^ *	422.0± 11.7^b^ ≠	607.6 ± 23.7^b^ ×	663.7± 26.4^c^ ×	318.0± 6.83^a^ ≠	
peri/fas of distal nerve section	0. 31 ± 0.03^a^		0. 30 ± 0.02^a^	0. 31 ± 0.02^a^	0.99 ± 0.10^c^ *		0.96 ± 0.13^c^ *	0.84 ± 0.06^a^ *	0.64 ± 0.04^b^ ×		0.63± 0.09^b^ ×	0.50 ± 0.10^a^ ×	0.55 ± 0.07^b^ *	0.56 ± 0.01^b^ *	0.48 ± 0.01^a^ *	0.59 ± 0.03^b^ ×	0.61 ± 0.07^b^ ×	1.18 ± 0.07^a^ ×	0.54 ± 0.01^b^ *	0.50± 0.02^ab^ *	0.42± 0.01^a^ ≠	

The letters are for comparison between groups at any time (week 2 green, week 4 red, and week 8 black), and the symbols (×, *, ≠) are for comparison between three different times in each group. The presence of non-similar letters or symbols indicates a significant difference (*P*<0.05)

**Figure 4:**
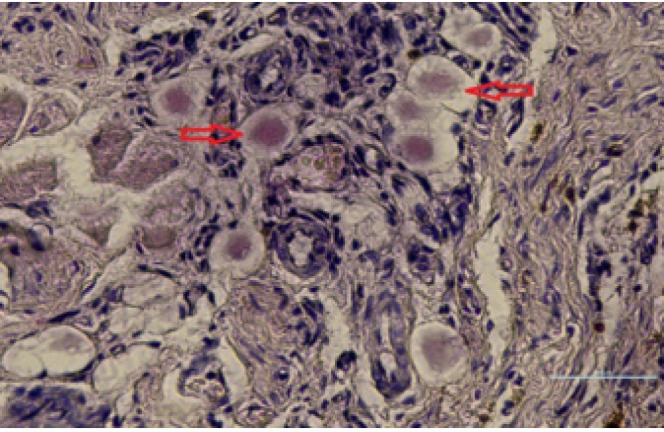
Cross section of spheroidal axons in the sciatic nerve of rats (arrows)

H&E staining, 400×

**Figure 5:**
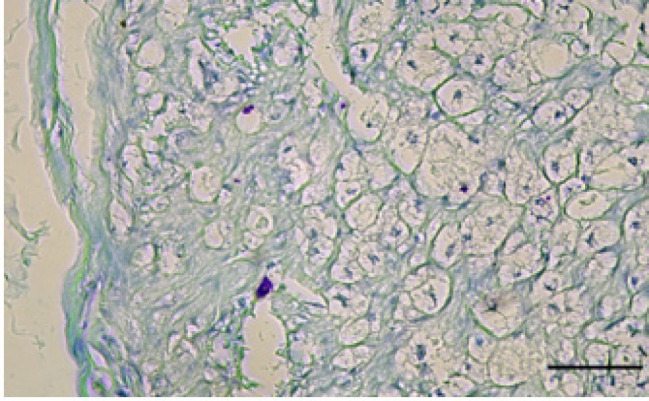
Tissue image shows cross sections of myelinated nerve fibers counted per unit area in the sciatic nerve of rats

Fast blue luxury staining, Bar 50 mµ

**Figure 6:**
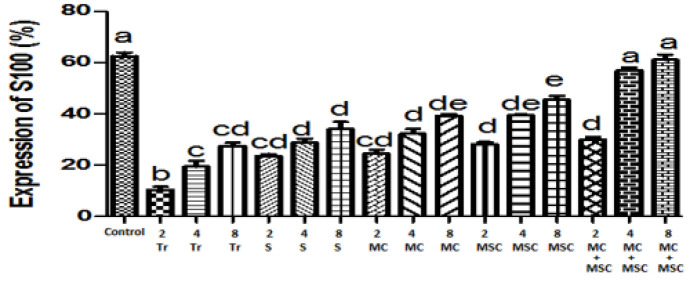
Comparison of mean S100 protein in the distal nerve of different groups of rats

The presence of non-similar letters indicates a significant difference (*P*<0.05)

**Figure 7:**
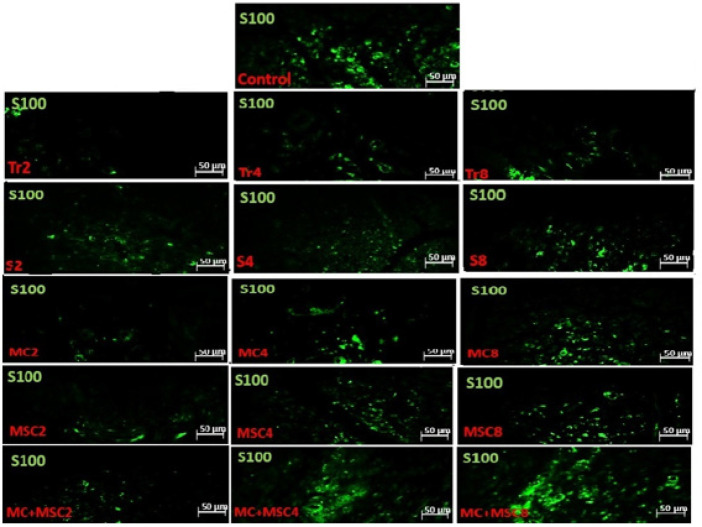
Fluorescent images related to the expression of S100 protein (green) in the distal section of transected sciatic nerve of different groups of rats

Immunohistochemical staining S100.

*The ratio of the number of muscle fibers nuclei to the number of muscle fibers (mn/mf)*

The results of this ratio in the second and fourth weeks showed a significant decrease in the Tr group compared to other groups (*P*<0.05), but in the fourth week, this decrease was not significant in the S group. In the 8th week, the control group with the highest ratio had a significant increase compared to the other groups (*P*<0.05), and the lowest ratio belonged to the Tr group (Table 1).

*The ratio of the number of blood vessels to the number of muscle fibers (v/mf)*

The v/mf in the second and fourth weeks was the lowest in the Tr group compared to the other groups (*P*<0.05). It increased in cell receiving and S groups, but not up to the control group and there was a significant difference between these groups with the control group (*P*<0.05). In the eight weeks, the cell and scaffold receiving groups had increased (v/mf) ratio but they were significantly different from the control group (*P*<0.05) (Table 1).

*The number of muscle mast cells*

In the present study, the mean number of mast cells in the fourth and eighth weeks in the Tr group showed a significant increase compared to other groups (*P*<0.05), and in the second week, there was no significant difference but only with the S group. In all weeks, the MC + MSC group had the lowest average number of mast cells among the other treatment groups (Table 2) (Figure 2).

*The percentage of sarcoplasmolysed muscle fibers*

The results of this study showed that the Tr group had the highest mean percentage of sarcoplasmolysis in all 3 time periods, which was not significantly different from the S group only in the second week (*P*<0.05). Furthermore, the use of scaffold and cells was able to reduce the rate of sarcoplasmolysis, especially in the MC + MSC group, which had a significant decrease in the eighth week compared to other groups (*P*<0.05) (Table 2) (Figure 2).

*The mean percentage of the number of necrotic muscle fibers*

Results obtained from the percentage of a number of necrotic fibers showed that this parameter was significantly increased in the Tr group compared to the other groups at 2, 4, and 8 weeks (*P*<0.05). The lowest percentage of necrotic fibers was observed in the MC + MSC group, which was significantly different from the other groups in the second and fourth weeks (*P*<0.05), and in the eighth week, there was no significant difference, but only with the MC group (Table 2) (Figure 2).

*The ratio of the Schwann cells number to the fibrocytes number (scn/fn) in the proximal nerve*

This study showed that the mean ratio of scn/fn in the Tr group decreased compared to the control group and had a significant difference (*P*<0.05), and these conditions were similar in all three time periods. However, the mentioned ratio in other groups was almost similar to the Tr group and had no significant difference with that group (Table 3).

*The ratio of Schwann cell number to fibrocyte number (scn/fn) at the site of nerve transection*

In the Tr group, there was the lowest number of scn/fn in periods of 2 to 8 weeks, while in other groups this ratio had a significant increase compared to the Tr group (*P*<0.05). In the second week, the groups in which the cell or scaffold was used had no significant differences from each other. In weeks 4 and 8, the MC + MSC group had a significant difference from the other intervention groups (*P*<0.05) and only in the eighth week were no significant differences with the MC group (Table 3).

*The mean percentage of myelinated nerve fibers number at the site of nerve transection*

This evaluation showed that there were no significant differences between Tr and intervention groups in the second and fourth weeks, while in the eighth week, there was a significant difference (*P*<0.05) (Table 3).

*Evaluation of nerve fascicle formation at the site of nerve transection*

The formation of nerve fascicles in 2 and 4-week periods was not observed in any groups except the MC + MSC group in the fourth week. Also, in the eighth week, nerve fascicles were formed, but only in cell groups, this formation was complete (Figure 3).

*The ratio of Schwann cells to fibrocytes (scn/fn) in the distal nerve*

The mean data obtained from the ratio of scn/fn showed a significant decrease in the tr group compared to other treatment groups in all three time periods (*P*<0.05) except the S group in the second week. However, the use of scaffold and cell in other groups increased this ratio, especially in the MC + MSC group which was significantly different in the eighth week from most intervention groups (*P*<0.05) (Table 4).

*The number of spheroid nerve fibers in the distal part*

The highest mean number of spheroid fibers showed in the Tr group so there was a significant difference with other groups (*P*<0.05). Also, the use of scaffold and cells at the site of transection reduced this mean number in all groups, and the MC + MSC group had the lowest mean number. These results were the same in all three time periods (Table 4) (Figure 4).

*The mean number of distal nerve mast cells*

Evaluation of this parameter showed a significant increase in 2 and 4 weeks in the Tr group, which also in the eighth week had a significant difference with other groups (*P*<0.05) except the MSC group. In Scaffold and cell receiving groups, the mean number of this parameter decreased, so that in the eighth week, the MC + MSC group had a significant decrease compared to other treatment groups (*P*<0.05) (Table 4).

*The mean Percentage of myelinated nerve fibers in the distal part*

The average percentage of myelinated fibers in the control group was higher than other groups in all time periods and showed a significant difference with them (*P*<0.05). However, there were no significant differences observed between the other groups (Table 4) (Figure 5).

*Mean number of myelinated nerve fibers per microscopic area unit in the distal part*

In this evaluation, increasing or decreasing number of myelinated nerve fibers in a fixed microscopic field indicates changes in their diameter. This means there was a significant decrease in the control group compared to the other groups in the second and fourth weeks, indicating the greater diameter of the fibers and their lower number per surface unit (*P*<0.05). In addition, the Tr group had a significant increase compared to the other groups in the eighth week (*P*<0.05). Also, in scaffold and cell groups the average number decreased by increasing the diameter of the fibers (Table 4) (Figure 5).

*Mean ratio of perineurium thickness to nerve fascicle thickness (* *per/fas* *) of distal nerve section*

The ratio in the Tr group increased significantly in the 2 and 4-week periods compared to other groups (*P*<0.05). Despite that, in the intervention groups, the ratio decreased and showed a significant difference with the control and Tr groups (*P*<0.05). Also, in the eighth week, no significant differences were observed between any of the groups (Table 4).

*Assay of S100 protein in the distal part of the nerve*

The results of this study on immunohistochemical staining showed that Schwann cell accumulation in the Tr group in the second and fourth weeks was significantly reduced compared to the other groups, and in the eighth week, only compared to the MSC and MC + MSC groups (*P*<0.05). Cell and scaffold receiving groups were also able to increase Schwann cell accumulation in the distal part, so that there was a significant difference in the MC + MSC group at 4 and 8 weeks with all treatment groups, and with the control group there was no significant difference (*P*<0.05). Furthermore, in all intervention groups, this parameter increased over time in all time periods of each group (Figures 6 and 7).

*Evaluation of muscle and nerve tissue parameters of each group at different time periods*

This study shows the changes of each group in three time periods which is evident in most groups, especially in the eighth week, as muscle and nerve tissue regeneration over time. The results are as follows:

1) The results obtained from the average thickness of muscle fibers confirmed a significant increase in the eighth week compared to other weeks in all groups (*P*<0.05) except the MSC group, which did not differ significantly from the second week despite the increase in the mean of the eighth week (Table 1).

2) The average showed that in most groups, there is no significant difference between the second and fourth weeks, but there is a significant difference with the eighth week (*P*<0.05). However, in the MC group, the eighth week was not significantly different from the second, and in the MC+MSC group, also the eighth week was not significantly different from the fourth (Table 1).

3) The results of mean mn/mf ratio, mean percentage of necrosis muscle fibers, ratio of scn/fn at the site of nerve transection, mean percentage of myelinated nerve fibers at the site of nerve transection, ratio of scn/fn in the distal nerve, and number of spheroid nerve fibers and mast cells in the distal part showed that in all groups the second and fourth weeks were not significantly different, but the eighth week was significantly different from the previous weeks (*P*<0.05) (Tables 1, 2, 3, and 4).

4) The ratio of vf/mf and the percentage of sarcoplasmolysis muscle fibers, in the eighth week of all groups had a significant difference with other weeks (*P*<0.05). However, the ratio of vf/mf in the Tr and MC groups and the percentage of sarcoplasmolysis muscle fibers in the Tr group in the second and fourth weeks were significantly different (*P*<0.05) (Tables 1 and 2).

5) The number of muscle mast cells decreased over time so that in all groups the eighth week had a significant decrease compared to the second and fourth weeks (*P*<0.05), but in the MC + MSC group, there was a significant difference only with the fourth week (*P*<0.05) (Table 2).

6) The ratio of scn/fn in the proximal nerve and ratio of peri/fas in the distal nerve section in most groups showed no significant difference between weeks 2, 4, and 8 (*P*<0.05). Despite that, the ratio of scn/fn in the eighth week of the MSC group had a significant increase compared to the other weeks and in the MC + MSC group in the eighth week, there was a significant difference only with the second week (*P*<0.05). Also, the ratio of peri/fas in the eighth week only in the MC + MSC group was significantly different from other weeks (*P*<0.05) (Tables 3 and 4).

7) The mean Percentage of myelinated nerve fibers in all groups except the S group and the mean number of myelinated nerve fibers per microscopic area unit in the distal part in all groups except the Tr group in the eighth week had a significant difference with the previous weeks (*P*<0.05) (Table 4). 

## Discussion

Denervation of the motor organs due to trauma causes muscle atrophy and movement problems (22). The present study aimed to provide a new solution in cell therapy for the rapid recovery of nerve fibers related to the sciatic nerve and related muscle. Motor nerve damage reduces the diameter of muscle fibers (23) 7 days after denervation in mice (24). In the present study, changes in the diameter of TCM fibers affected by sciatic nerve transection showed that the cell therapy in the time-dependent pattern and scaffold in the eighth week had a better improvement compared to previous weeks. Muscle immobility (following denervation) reduces satellite cells (25) and with atrophy, increases connective tissue and fibrosis (26). In the present study, the mean ratio of mn/fn in the fourth and eighth weeks was significantly reduced in the Tr group while in the scaffold and cellular groups, the healing process was accelerated due to time-dependent nerve repairing. Electrical stimulation of denervated muscle increases the nuclei of muscle fibers and the formation of embryonic muscle fibers MHC + (as an indicator of muscle regeneration) by accelerating the differentiation of satellite cells (27). The study of the mean mn/mf ratio showed an increase, especially in the hybrid cell group which could be due to the role of MCs in angiogenesis around the repairing nerve fiber (28) as well as the neurogenic role of these cells (29) and their cooperation with mesenchymal cells (13) in their differentiation process into the Schwann-like cells (30). In denervated skeletal muscles, atrophy of muscle fibers and fibrosis occur along with changes in the vascular bed. In one study, 10 days after sciatic nerve transection, 20% of capillaries decreased, and 13% increase occurred during the regeneration process on days 20-30 (31). In this regard, the present study showed that the mean of v/mf in the Tr group compared to other groups was minimal, while in all weeks, it increased due to the improvement of neural flow in S and cellular groups. Most blood vessels produce signals such as Artemide and neurotrophin 3 so that the axons are placed in the path of the arteries, and nerves also produce signals such as vascular endothelial growth factor to stimulate vascular growth (32), so when the nerves are damaged, the arteries in their path are also reduced. Mast cells proliferate during inflammation and tissue damage and have the potential to degenerate myofibers through protease secretion (33). Studies on denervated muscles or myopathy have also confirmed the accumulation of mast cells in the endomysium of the muscle (34). In this study, the lowest mean number of these cells was seen in the control group and then the MC + MSC group. This finding showed that the use of cells or scaffolds, especially hybrid cells, by modulating the repair of transected nerves, improved cellular reactions in muscle tissue. During damage to a muscle following denervation, muscle fibers are completely decomposed by myolysis (35), by the activation of proteases, and subsequent muscle degeneration (36). But in sarcoplasmolysis, the sarcoplasm becomes eosinophilic (hyalinate) with the muscle striation disappearing while the sarcolemma is preserved (37). The findings of this study showed that sarcoplasmolysis in the Tr group was more or almost equal to the other groups. However, in relation to the combined cell group, it decreased significantly at week 8 which shows the synergistic effect of mesenchymal and mast cells on nerve and muscle regeneration. Muscle denervation can lead to irreversible structural damage like necrosis of muscle fibers (38) and then with the destruction of the sarcolemma and the pyknosis of nuclei, the cytoplasm of fibers becomes eosinophilic (39). In the present study, the rate of muscle necrosis showed the highest and lowest percentages in the Tr and MC + MSC groups respectively. In a study examining the soleus muscle after sciatic nerve transection, many necrotic foci were seen in the second week after denervation but in the eighth week, there was a lot of improvement in muscle (40). As mentioned earlier, when nerves are damaged, the arteries in their pathway also decrease. Therefore, it can be concluded that one of the most important causes of muscle necrosis is a significant reduction in the number of vessels feeding the muscle following the interruption of nerve conduction. In scn/fn evaluation in the proximal nerve, the experimental groups did not show significant changes compared to each other, but in general, this ratio decreased compared to the control group. Therefore, it can be concluded that denervation had fewer negative effects on the proximal part and proximal degeneration (41) reduced Schwann cells and caused fibrosis in the groups compared to the control group. The number of myelinated nerve fibers at the site of nerve transection is directly related to the rate of nerve regeneration (42). In the present study, the ratio of scn/fn at the site of transection was higher in the MC + MSC group compared to other groups which indicates the improvement of fibrosis. The results of a study also reported the effect of mesenchymal cells of amniotic fluid in reducing fibrosis at the site of rat sciatic nerve crush (43). Also, the increase of S100 protein in the distal part of the transected sciatic nerve in cellular groups, especially the MC + MSC group, due to the increase of Schwann cells and myelinating of fibers is a reason for the reduction of fibrosis in these groups. In a study, the number of myelin fibers increased at a distance of 5 mm from the site of sciatic nerve crush and transection using amniotic mesenchymal cells (44). In addition to neurogenic secretions of mast cells (29) and mesenchymal cells (11), the stimulatory effects of mast cells on the differentiation of mesenchymal cells (13) into Schwann-like cells (30) confirm the increase in this ratio in cellular groups, especially hybrid cell groups. In the regeneration of peripheral nerve transection, the use of cell therapy techniques to form the endoneurium and perineurium of the nerve is very important because perineurium as a blood-nerve barrier prevents damage to nerve endings (45). The present study on the formation of nerve fascicles at the site of nerve transection showed that at the eighth week in all groups, nerve fascicles were forming; while in cellular groups the fascicles were almost completely formed, and in the MC + MSC group even in the fourth week the complete formation of fascicles was observed. These results indicated the effect of using mast cells in the production of important factors in nerve repair, including NGF, neuropeptide, endothelin-1 (29), and secretion of nerve regenerating factors by bone marrow-derived mesenchymal cells such as NGF, BDNF, ciliary neurotrophic factor (CNTF), glial cell neurotrophic factor (GDNF), CNTF, and neurotrophin-3 (NT-3) (11) over 8 weeks. S100 protein is a group of low molecular weight acidic proteins found in glia and Schwann cells that play a regenerative role in the rat nervous system (46). Schwann cell proliferation is the primary nerve response to injury (47), in which Schwann cells proliferate to replace dead cells (48). Findings from the S100 protein assay showed that in the intervention groups, the mean density of Schwann cells at the distal end increased compared to the Tr group this increase was especially evident in the eighth week and showed the positive effect of the time parameter. Also, due to the synergistic effect of mast cells and mesenchymal cells, Schwann cell density was highest in the combined cell group at weeks 4 and 8, which increased in the control group. In this regard, the data obtained from scn/fn in the distal nerve had similar results. Also, because Schwann cells act as a precursor to nerve repair in the nerve regeneration process, they create a nerve growth channel (49). Therefore, the results of this study showed the positive effect of cell therapy on the nerve regeneration process. Many studies have shown Schwann cell proliferation after the use of mesenchymal cells in neurological injuries (42) and an increase of S100 protein in models of transection or crush of the sciatic nerve that was in the process of repair (48). Due to the role of mast cells in stimulating the differentiation of mesenchymal cells (13), increasing the amount of S100 protein, especially in the combination cell group is completely consistent with the results of previous studies. Focal swelling of axons or axonal spheroids in many CNS neurodegenerative diseases (50), distal nerve area in CNS injuries (51), and Wallerian degeneration are also observed (50). Spheroid axons are seen in the CNS 6 hr after axotomy and 37–44 hr after nerve injury in the PNS (52). Counting the number of spheroid nerve fibers in the present study showed that the use of scaffold and cell therapy reduced the spheroid fibers in the distal part of the transected nerve and this effect was more in the group of combined cells than others. Mast cells increase in neurogenic inflammation (53) as well as at the neuromuscular junction following nerve injury (34). Accumulation of these cells in nerve injuries has the potential to initiate damage to motor nerves, Schwann cells, and the capillary network, directly or indirectly (34). Evaluation of the average number of mast cells around the distal part of the nerve indicates the amount of inflammation in this area, which showed the highest average in the Tr group; and among the intervention groups, the MC + MSC group had the lowest mean. Morphometric reports from sciatic nerve injuries also indicate a lack of concomitant histomorphometric changes (54). So in one study, the density of nerve fibers stabilized at 3 months after sciatic nerve transection, but myelination was still increasing at 12 months (54). Other studies have shown that myelination of nerve fibers is not complete until 12 weeks after denervation (55). Since the expression of S100 protein reflects the degree of myelination of nerve fibers and the rate of S100 immune reactions is related to the thickness of the myelin sheath of Schwann cells (46), the decrease in the number of myelin fibers per microscopic surface unit (due to the increase in the thickness of the myelin sheath) over time is directly related to the increase in S100 protein (Schwann cells) in weeks 4 and 8 of the present study. These results are due to the effect of mediators released from mast cells on the differentiation of mesenchymal cells (13) to Schwann-like cells (12) as well as the angiogenic effects of some other mast cell mediators (56) to provide suitable conditions for nerve growth. Also, in many studies, the differentiation capacity of bone marrow mesenchymal cells into Schwann cells and an increase in the myelin thickness of nerve fibers was reported (47). Abnormal perineurium fibrosis is a proliferation of fibroblasts, which leads to excessive deposition of type 1 collagen in peripheral nerve injuries (57). Based on the findings of the present study, the mean of peri/fas in the distal part of the Tr group in weeks 2 and 4 was higher than the other groups, while in the other intervention groups, this ratio decreased. Scar formation in nerve tissue is associated with a reduction in the number of nerve fibers at the site of injury and inhibition of regeneration (58). In fact, increasing the thickness of the epineurium and perineurium delays the regeneration of nerve injuries (58). Numerous studies have confirmed the anti-scar effect of various drug agents on the healing of peripheral nerve injuries (59) and the sciatic nerve (60). Also, the study of parameters related to the evaluation of the nerve and TCA of each group separately at different times showed the improvement of tissue conditions over time, especially in the eighth week compared to previous weeks in most groups.

## Conclusion

Overall, the results of the present study showed that the histomorphometric and histopathological changes of the TCM denervation in the intervention groups and especially in the MC + MSC group improved in various parameters. In the proximal section of the transected nerve, due to the trophic relationship between the peripheral and central part of the nerve minor histopathological changes were observed. At the transection site, the connection between the proximal and distal sections was more completely established in the eighth week, but it has been seen that the myelination of fibers requires more time. In the distal part, the results of histopathological evaluation showed the process of improvement of the parameters was better in the mixed cells group. According to the results of this study, co-implanted mast cells and mesenchymal cells at the site of nerve transection have a synergistic function with each other. In addition to stimulating angiogenesis, mast cells may differentiate mesenchymal cells into Schwann cells, which accelerates nerve regeneration and improves affected muscle (TCM).
